# *Clostridium scindens*: a human gut microbe with a high potential to convert glucocorticoids into androgens[S]

**DOI:** 10.1194/jlr.M038869

**Published:** 2013-09

**Authors:** Jason M. Ridlon, Shigeo Ikegawa, João M. P. Alves, Biao Zhou, Akiko Kobayashi, Takashi Iida, Kuniko Mitamura, Genzoh Tanabe, Myrna Serrano, Ainee De Guzman, Patsy Cooper, Gregory A. Buck, Phillip B. Hylemon

**Affiliations:** * Department of Microbiology and Immunology and Virginia Commonwealth University, Richmond, VA 23298; ** Center for Biological Complexity, Virginia Commonwealth University, Richmond, VA 23298; † Veterans Affairs McGuire Medical Center, Richmond, VA 23249; § Faculty of Pharmaceutical Sciences, Kinki University, Kowakae, Higashi-Osaka 577-8502, Japan; †† Department of Chemistry, College of Humanities & Sciences, Nihon University, Sakurajousui, Setagaya, Tokyo 102-0074, Japan; and; §§ Department of Biology, University of Virginia, Charlottesville, VA 22903

**Keywords:** RNA-Seq, microbiome, steroid

## Abstract

*Clostridium scindens* American Type Culture Collection 35704 is capable of converting primary bile acids to toxic secondary bile acids, as well as converting glucocorticoids to androgens by side-chain cleavage. The molecular structure of the side-chain cleavage product of cortisol produced by *C. scindens* was determined to be 11β-hydroxyandrost-4-ene-3,17-dione (11β-OHA) by high-resolution mass spectrometry, ^1^H and ^13^C NMR spectroscopy, and X-ray crystallography. Using RNA-Seq technology, we identified a cortisol-inducible (∼1,000-fold) operon (*des*ABCD) encoding at least one enzyme involved in anaerobic side-chain cleavage. The *des*C gene was cloned, overexpressed, purified, and found to encode a 20α-hydroxysteroid dehydrogenase (HSDH). This operon also encodes a putative “transketolase” (*des*AB) hypothesized to have steroid-17,20-desmolase/oxidase activity, and a possible corticosteroid transporter (*des*D). RNA-Seq data suggests that the two-carbon side chain of glucocorticords may feed into the pentose-phosphate pathway and are used as a carbon source. The 20α-HSDH is hypothesized to function as a metabolic “rheostat” controlling rates of side-chain cleavage. Phylogenetic analysis suggests this operon is rare in nature and the *des*C gene evolved from a gene encoding threonine dehydrogenase. The physiological effect of 11β-OHAD on the host or other gut microbes is currently unknown.

In the intestinal ecosystem, microbes have evolved enzymes to modify a number of host bile acids and steroids (1, 2). During the enterohepatic circulation (EC), bile salts are synthesized in the liver, concentrated in the gallbladder, and function in the lumen of the small intestine to absorb dietary lipids and limit microbial growth at the site of nutrient uptake (3). Most of these bile salts are actively transported from the lumen of the ileum into the portal circulation and back to the liver; however, roughly 600–800 mg of bile salts escape into the cecum where they are rapidly deconjugated to free bile acids (1, 3). Bile acid 7α/β-dehydroxylating bacteria are organisms capable of converting primary bile acids made by the host to harmful secondary bile acids, deoxycholic acid, and lithocholic acid (1). These bacteria normally comprise a small proportion of the gut microbiota (∼10^3^–10^4^/g wet weight) and consist of species within the genus *Clostridium* (4).

Host glucocorticoids which enter the large bowel are metabolized by at least one isolate of bacteria with bile acid 7α-dehydroxylating activity (5). The original isolate, *Clostridium* strain “19”, had the capacity to cleave the side chain from 21α,17α-hydroxy glucocorticoids, converting them into C-19 androgens (5, 6). *Clostridium* strain 19 was later named *Clostridium scindens* (type strain American Type Culture Collection (ATCC) 35704) which means “to cut” (6). Previously, our lab purified and characterized an NAD(P)^+^-dependent 20α-hydroxysteroid dehydrogenase (HSDH) from cell extracts of *C. scindens* ATCC 35704 in addition to a partially purified steroid-17,20-desmolase (SDase); however, the genes encoding these enzymes have yet to be identified (7, 8).

In mammalian cells, side-chain cleavage of glucocorticoids proceeds by an oxygen-dependent P450 monooxygenase reaction (9). However, gut microbial SDase occurs under anaerobic conditions. We showed previously that SDase was optimal under anaerobic conditions (optimum circa −130 mV) (6). Therefore, we expect that the mammalian and gut microbial steroid-17,20-desmolase gene(s) to have formed by convergent evolution. Human 20α-HSDH is encoded by AKR1C1, a member of the aldo/keto reductase family (10). However, AKR1C1 and microbial 20α-HSDH show different substrate specificities, again suggesting possible differences in gene ontology (7, 8). The genome of *C. scindens* ATCC 35704 has recently been sequenced as part of the Human Microbiome Project reference gut microbial genome initiative (Accession: PRJNA18175). Both 20α-HSDH and steroid-17,20-desmolase activities in *C. scindens* ATCC 35704 are cortisol inducible (7, 8). We decided to adopt a genome-wide transcriptomics (RNA-Seq) approach for gene discovery given that we can control induction of the genes for cortisol metabolism, the genome sequence can be used as a scaffold to organize transcripts, and the mechanism for SDase and thus the gene ontology is unknown, and not obvious from genome sequence alone.

In the present report, we thoroughly characterize the end product of bacterial steroid-17,20-desmolase and verify the identity of this steroid metabolite as 11β-hydroxyandrosten-3,17-dione (11β-OHA) through mass spectrometry (MS), ^1^H and ^13^C nuclear magnetic resonance (NMR) spectroscopy, and X-ray crystallography. Next, we identify a cortisol-inducible operon (*desABC*D) by quantifying and comparing mRNA levels from cells induced by cortisol versus uninduced cells of *C. scindens* ATCC 35704 using RNA-Seq. We demonstrate that this operon encodes a 20α-HSDH (*des*C) with substrate specificity similar to that reported for the native enzyme. Bioinformatic analysis suggests a hypothesis for the mechanism of microbial steroid-17,20-desmolase (*des*AB). The steroid-17,20-desmolase is hypothesized to proceed by transketolation with the two-carbon side chain feeding into the pentose-phosphate pathway. Our data also show that SDase activity is rare among bile acid 7α-dehydroxylating isolates from the human gut, and that only a fraction of *C. scindens* isolates have this activity. Finally, we perform thorough phylogenetic analysis of the 20α-HSDH gene product (DesC), which appears to be rare in the gut microbiota of most organisms. The gene appears to have evolved from the gene encoding threonine dehydrogenase and the operon appears to be conserved in organization, though not in function, among a small number of anaerobic microbes from diverse environments.

## MATERIALS AND METHODS

### Purification and identification of steroid-17,20-desmolase product

Cortisol reaction products were extracted from reaction buffer by 1/10 vol 1 M HCl followed by two extractions with 2 vol of methylene chloride, dried under nitrogen, and separated by silica B gel TLC plates with solvent A [5:25:0.2 isooctane:ethyl acetate:glacial acetic acid (v/v/v)] (6). Products were scraped from TLC plates and extracted from silica with ethyl acetate, dried under nitrogen, and resuspended in methanol. Products were further purified by reverse-phase HPLC on a Beckman ODS C_18_ 10 × 250 mm semi-prep column. Samples were separated at 2.5 ml/min at 25°C and monitored at 240 nm. Peaks were collected and dried under a nitrogen gas atmosphere.

### High-resolution mass spectrometry

High-resolution mass spectrometry (HR-MS) using an atmospheric pressure chemical ionization (HR-APCI-MS) was carried out using a JEOL AccuTOF JMS-T100LC liquid chromatograph-mass spectrometer (JEOL, Tokyo, Japan) with an APCI source and coupled to an Agilent 1200 series binary pump (Agilent, Santa Clara, CA) in the negative ion mode. HR-APCI-MS of the sample was carried out in the flow injection mode, using methanol as the mobile phase at a flow rate of 1 ml/min. The ionization conditions were as follows: needle voltage, −2 kV; ion guide peak voltage, 2.5 kV; ion source temperature, 80°C; desolvation temperature, 500°C; orifice 1, 2 and 3 voltages, −85, −5, and −15 V respectively; mass range, *m/z* 50–1,000; nebulizing gas, N_2_.

### NMR spectroscopy

^1^H and ^13^C NMR spectra were obtained on a JNM-ECA 800 instrument operated at 800 and 200 MHz, respectively, with CDCl_3_ containing 0.1% tetramethylsilane (TMS) as the solvent. Chemical shifts were expressed in δ (ppm) relative to TMS, and the following abbreviations are used: s, singlet; d, doublet; br, broad. The ^13^C distortionless enhancement by polarization transfer (135°, 90°, and 45°) spectrum was measured between CH_3_, CH_2_, CH, and coherence based on their proton environments. In order to further confirm the ^1^H and ^13^C signal detected heteronuclear multiple quantum (^1^H-^13^C coupling) and ^1^H detected heteronuclear multiple bond correlation (long-range ^1^H-^13^C coupling) experiments were also performed.

### X-ray crystal structure determination

Colorless crystals of steroid-17,20-desmolase reaction product suitable for X-ray crystal structure determination were grown by recrystallization of the compound from ether-*n*-hexane gave colorless needles with a melting point of 197–199°C. A crystal having dimensions of 0.25 × 0.10 × 0.05 mm^3^ was selected. The X-ray intensity measurements were carried out on a Rigaku Micro7HFM-VariMax Saturn 724R CCD system with a confocal X-ray mirror, using graphite-monochromated Mo Kα radiation (λ = 0.7107 Å) at 220 K. The structure was solved using direct methods and refined by a full-matrix least-squares procedure based on *F*^2^ using the CrystalStructure crystallographic software package. The crystal structures were refined with anisotropic temperature factors for all nonhydrogen atoms. The positions of hydrogen atoms were generated theoretically, and were refined using the riding model. Crystallographic details are summarized in supplementary Table II.

### Bacterial strains, growth conditions, and sterol induction

*C. scindens* ATCC 35704 was purchased from ATCC. *C. scindens* ATCC 35704 was stored as a 30% glycerol stock at −80°C prior to this study, and working stocks were cultivated in chopped meat broth. After overnight growth in Brain Heart Infusion broth (BHI), a 5% inoculum was transferred to 100 ml BHI containing 50 μM cortisol. Cells used for RNA isolation were cultivated to O.D. _600 nm_ of 0.4 (mid-log phase), and quenched in a 1:2 v/v solution of RNALater (Ambion, Grand Island, NY) reagent and kept overnight at 4°C. Cells were pelleted the following day by centrifugation and stored until processed at −80°C. Induction was verified by HPLC assay (see below). *Escherichia coli* BL21 (DE3) RIL was purchased from Agilent Stratagene (Santa Clara, CA). *E. coli* was cultivated in Luria-Bertani medium containing appropriate antibiotics. Steroids and bile acids were purchased from Sigma-Aldrich (St. Louis, MO) and Steraloids (Newport, RI).

### HPLC assay for steroid-17,20-desmolase activity

An HPLC assay was used to verify steroid-17,20-desmolase activity in *C. scindens* ATCC 35704 before proceeding with RNA isolation. This assay was also used to determine whether other fecal isolates with bile acid 7α-dehydroxylating activity also possess steroid-17,20-desmolase activity. Strains exhibiting bile acid 7α-dehydroxylating activity were isolated from human volunteer stool as described previously (11). We utilized an HPLC assay to determine whether strains of bile acid 7α-dehydroxylating bacteria have steroid-17,20-desmolase activity. Cortisol and 11β-hydroxyandrostenedione each absorb at 240 nm owing to their 3-oxo-Δ^4^ structures. Once late log phase was reached, steroids were extracted from the medium with dichloromethane, dried, resuspended in methanol, and separated on an Agilent Eclipse C_18_ column (5 μm, 4.6 × 250 mm) integrated on an Agilent 1200 series HPLC. The column temperature was maintained at 40°C, the flow rate was 1 ml/min, and the solvent was 50% methanol in HPLC grade water (Sigma-Aldrich). Concentration was determined by peak area compared with a standard curve of peak area versus known concentrations of cortisol and 11β-hydroxyandrostenedione.

### Purification of mRNA by magnetic bead-capture hybridization

Cells were quenched with RNALater solution (Ambion) and stored at −80°C until processing. Total RNA was recovered from cells following disruption by bead beating in the presence of acid phenol. RNA was treated several times with DNase to remove contaminating genomic DNA, and was further purified using the MEGAclear Kit (Ambion). mRNA was enriched by magnetic-bead capture hybridization using custom biotinylated TEG-spaced oligonucleotides designed along the length of the 16s rRNA and 23s rRNA molecules from *C. scindens* ATCC 35704 (supplementary Table I).

Cells were stored at −80°C in RNAlater solution (Ambion). Cells were then collected by centrifugation and washed with lysis buffer (200 mM NaCl, 20 mM EDTA, diethylpyrocarbonate-treated water) and collected again by centrifugation. Cells were resuspended in 500 μl lysis buffer and transferred to 2 ml screw-cap bead-beating tubes (Sarstedt, Germany) to which 200 μl zirconium beads, 210 μl 20% SDS solution (Ambion), and 1 ml 5:1 acid phenol were added. Cells were then disrupted on a Mini-BeadBeater (Biospec Products, Inc., Bartlesville, OK) at maximum speed twice for 1 min, with tubes kept on ice in between treatments. The aqueous and phenol phases were then separated by centrifugation, and the aqueous phase was washed once with 1 ml 5:1 acid phenol. Nucleic acids in the aqueous phase were then precipitated at −80°C for 20 min by addition of 1/10 vol 5 M ammonium acetate (Ambion), 1 μl Glycoblue (Ambion), and 1 vol ice-cold isopropanol, followed by centrifugation at 13,600 *g* for 20 min. RNA (>200 bp) was then purified using the MEGAclear kit (Ambion) according to the manufacturer's instructions. Contaminating genomic DNA was removed by treatment with TURBO DNase (Ambion) according to the manufacturer's instructions. MEGAclear and DNase treatment steps were repeated once. At this point, RNA purity and integrity was checked spectrophotometically by the A_260_:A_280_ ratio, the range of pure RNA falling between 1.7 and 2.1, and integrity checked by separating RNA on a 1.6% denaturing agarose gel. RNA samples with distinct 23S rRNA and 16S rRNA bands were further purified. In addition, residual genomic DNA contamination was determined by RT-PCR using the RT-for-PCR kit (Clontech) and 200 ng purified RNA.

Dynabeads M-280 Streptavidin (Invitrogen, Grand Island, NY) was made RNase-free according to the manufacturer's recommendations. Biotinylated oligonucleotides were bound to beads (5 mg) (through streptavidin) by resuspending in 500 μl diethylpyrocarbonate-treated 0.5× SSC (0.075 M NaCl; 0.0075 M trisodium citrate dehydrate; 10 mM Tris, pH 7.0; 1 mM EDTA) containing 360 pmol each biotinylated oligonucleotide (denatured by heating to 90°C for 5 min and cooling on ice for 3 min). Beads were then captured on a Promega magnetic stand (Promega, Madison, WI), the supernatant containing unbound probes aspirated, and residual unbound probes washed twice with one volume 0.5× SSC, and twice with 6× SSC (0.9 M NaCl; 0.09 M trisodium citrate dehydrate; 10 mM Tris, pH 7.0; 1 mM EDTA). Efficiency of binding was determined by comparing A_260 nm_ of oligo-mix after bead incubation and washing and comparing to oligo-mix without bead incubation. Capture-hybridization was performed on a Biorad C1000 thermocycler (Biorad, Hercules, CA). First, 1 μg total RNA suspended in 35 μl 6× SSC in a RNase-free 0.2 ml PCR tube was heated to 70°Cfor 5 min to denature RNA, followed by 0°C for 3 min at which point the thermocycler was paused allowing addition of 150 μg oligo-bound beads. The thermocycler was resumed at a temperature of 68°C for 30 min allowing capture hybridization between rRNA molecules and oligo-bound beads. Beads were then captured on a magnetic stand, and the supernatant was combined with two additional (1 vol) washes with 6× SSC, and the enriched mRNA was centrifuged and magnetically captured to remove residual beads before being precipitated. RNA bound to beads was quantified by A_260 nm_ after removal by three stringent washes with 0.5× SSC containing 10% formamide at 70°C. Enriched mRNA was quantified on a NanoDrop 2000 (Thermo Scientific, Waltham, MA) and stored at −80°C until sequencing.

### Whole transcriptome sequencing

MRNA-enriched samples were processed using the Roche cDNA synthesis system following the manufacturer's protocol. Resulting cDNA libraries were sequenced in a Roche 454 GS FLX Titanium system (Roche, Indianapolis, IN) using the rapid library protocol according to the manufacturer's instructions. The sequencing yield was of ∼300,000 reads for the libraries derived from cortisol-induced cells and from uninduced control cells.

Remaining ribosomal and transfer RNA reads were removed from the sequence dataset by BLAST searches (12) of these reads against a database of known *Clostridium* rRNA and tRNA sequences, using a minimum 90% identity cutoff. Non-rRNA reads were then mapped to the *C. scindens* ATCC 35704 genome using Burrows-Wheeler transformation (13), and resulting alignments were analyzed by CuffLinks (14) for differential expression detection.

### Cloning and overexpression of *desC* gene in *E. coli*

Genomic DNA was isolated from *C. scindens* ATCC 35704 by enzymatic treatment, followed by bead beating as described previously (15). A streptavidin tag was engineered into the reverse primer and the *des*C gene was PCR amplified, restriction digested, and ligated into the expression vector pSport1.

Primer sequences were as follows: pSportCTSBPEDS07887.1F, 5′-AAGCTTTTGTTAGATATCAAGGAGGAAAAAAAAATGAGAC-3′; and pSportCTSBPEDS07887.1R, 5′-GAGCTCTTATTTTTCGAACTGCGGGTGGCTCCATTCGT CCATCTTAATTACGATCTTTGC. PCR was performed using the TITANIUM Taq kit (Clontech) on a Biorad C1000 thermocycler. PCR products and pSport1 were then cut with the appropriate restriction enzymes (New England Biolabs, Ipswich, MA) and ligated with T4 DNA ligase (New England Biolabs). Plasmids were transformed into *E. coli* DH5α by heat shock at 42°C and cultivated for 1 h in Super Optimal Broth medium (Invitrogen) before plating on LB ampicillin (100 μg/ml). Both strands of the insert were sequenced using T7 promotor and terminator primers. The protein was overexpressed in *E. coli* BL21(DE3)RIL and purified by Strep-Tactin affinity chromatography. Colonies were grown in LB ampicillin and induced for overexpression by addition of Isopropyl β-D-1-thiogalactopyranoside to 1 μM final. Cultures were screened for expression by Western blot hybridization with the Strep Tag II antibody (IBA, Kansas City, MO). Cell extracts were prepared by treating cells with 5 μg/ml lysozyme in buffer A (20 mM sodium phosphate buffer, pH 7.0; 0.1 M NaCl; 20% glycerol; 10 mM 2-mercaptoethanol) on ice for 1 h followed by two passes through a French pressure cell at 1,500 psi. Cell extract was then centrifuged for 30 min at 16,000 *g*. The supernatant was then applied to a Strep-Tactin affinity column equilibrated with buffer A. The column was washed with buffer A until protein was not detected in the eluent. Recombinant proteins were eluted in buffer A containing 2.5 mM desthiobiotin.

### 20α-HSDH enzyme assay

20α-HSDH activity was measured by continuous spectrophotometric assay at 340 nm performed in buffer A containing 50 μM cortisol (Steraloids, Inc., Newport, RI) and 150 μM NADH. A molar extinction coefficient of 6,220 M^−1^cm^−1^ was used to determine product concentration. Reaction rates were performed under varying substrate concentrations below and above the respective *K*_m_ values. Initial linear region of the reaction progress curves was utilized for determining the reaction rates. *K_m_* and *V_max_* values were determined by Lineweaver-Burk plots of the data.

### Phylogenetic analyses of *desC*

Putative orthologs of *desC* were identified by BLAST (9) search of the *C. scindens* ATCC 35704 protein sequence against the National Center for Biotechnology Information Non-redundant protein database, accepting no more than 10,000 matches and employing a maximum E-value cutoff of 1e-20. The resulting sequence set was then filtered to contain at most one strain per species and to remove duplicate sequences and those that were too long or too short, defined respectively as being longer than 1.5 times or shorter than 0.5 times the average sequence length (356 amino acids) of the original sequences. To save computational time, the resulting set of 2,045 protein sequences was submitted to a two-step phylogenetic analysis. For both steps, sequences were aligned using MUSCLE version 3.8.31 (16) and submitted to maximum likelihood analysis using RAxML version 7.2.8 (17); the substitution model employed was WAG (18) using empirical residue frequencies and the γ distribution model of rate heterogeneity (model PROTGAMMAWAGF). The first step consisted of one detailed tree search, in order to get a first overview of phylogeny and identify the region where *C. scindens*’ *desC* and its nearest neighbors would be located. The 341 sequences from the resulting subtree containing *C. scindens*’ *desC* and its nearest neighbors plus a few outgroups from another smaller subtree were then submitted to the thorough maximum likelihood tree searches. We performed bootstrap analysis with 100 pseudoreplicates, and bipartition frequencies were then drawn on the best tree out of 20 independent tree searches. Trees were drawn and formatted using TreeGraph2 (19) and Dendroscope (20), and further cosmetic adjustments were done in the Inkscape vector image editor. As a reference species tree of the clostridia, we used the automated phylogenomic analysis from PATRIC, which shows *C. scindens* as distantly related to most other *Clostridium* species, being deeply embedded in the Lachnospiraceae family and with several *Ruminococcus* species in close phylogenetic proximity. The *Clostridium* genes identified as closely related to *C. scindens*’* desC* belong to species only distantly related to this cluster of *C. scindens*, *Ruminococcus*, and the Lachnospiraceae. However, no member of the latter two taxa presents a gene closely related to *C. scindens*’ *desC*. On the other hand, *desC* grouped with a group that, besides the clostridia (*Clostridium leptum* DSM 753 ZP_02081557, *Clostridium ljungdahlii* DSM 13528 YP_003780744 and YP_003780741, *Clostridium* sp*.* MSTE9 EJF39056, and *Clostridium carboxidivorans* P7 ZP_05390048), also contained a sequence from the Spirochaetes *Treponema primitia* ZAS-1 (ZP_09717055) and the actinobacteria *Modestobacter marinus* (YP_006366820); sequences in this group were very similar to each other, with BLAST E-values of around 1E-50 or better in comparison to *C. scindens*’ *desC*. The sister group for this clade is composed of two *Thermotoga* species (*Thermotoga maritima* MSB8 NP_228110 and *Thermotoga petrophila* RKU-1 YP_001244210) and a group of Metazoa (15 insects, and one each of Cnidaria, Cephalochardata, and Tunicata); in this case, similarity compared with *desC* was much lower, with values between 1E-35 and 1E-21. The whole assemblage described above forms a clade with the very high bootstrap support value of 97, and is not closely related to other Firmicutes bacteria present in the tree.

## RESULTS

### Identification of the side-chain cleavage product of cortisol by *C. scindens* ATCC 35704

**Fig. 1.**
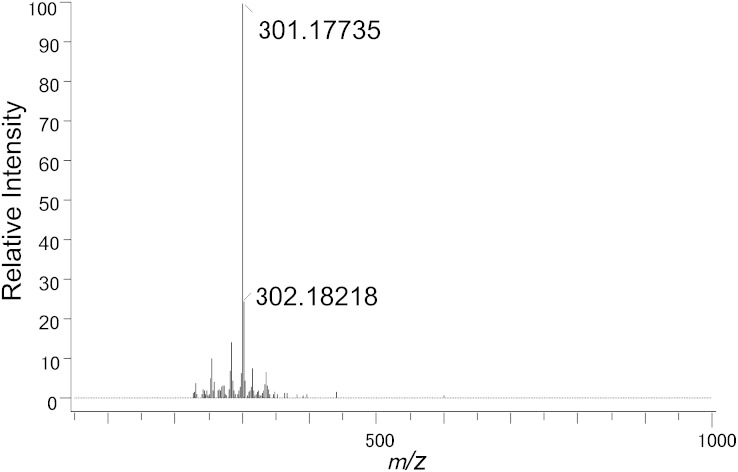
HR-APCI-MS of steroid-17,20-desmolase reaction product.

**Fig. 2.**
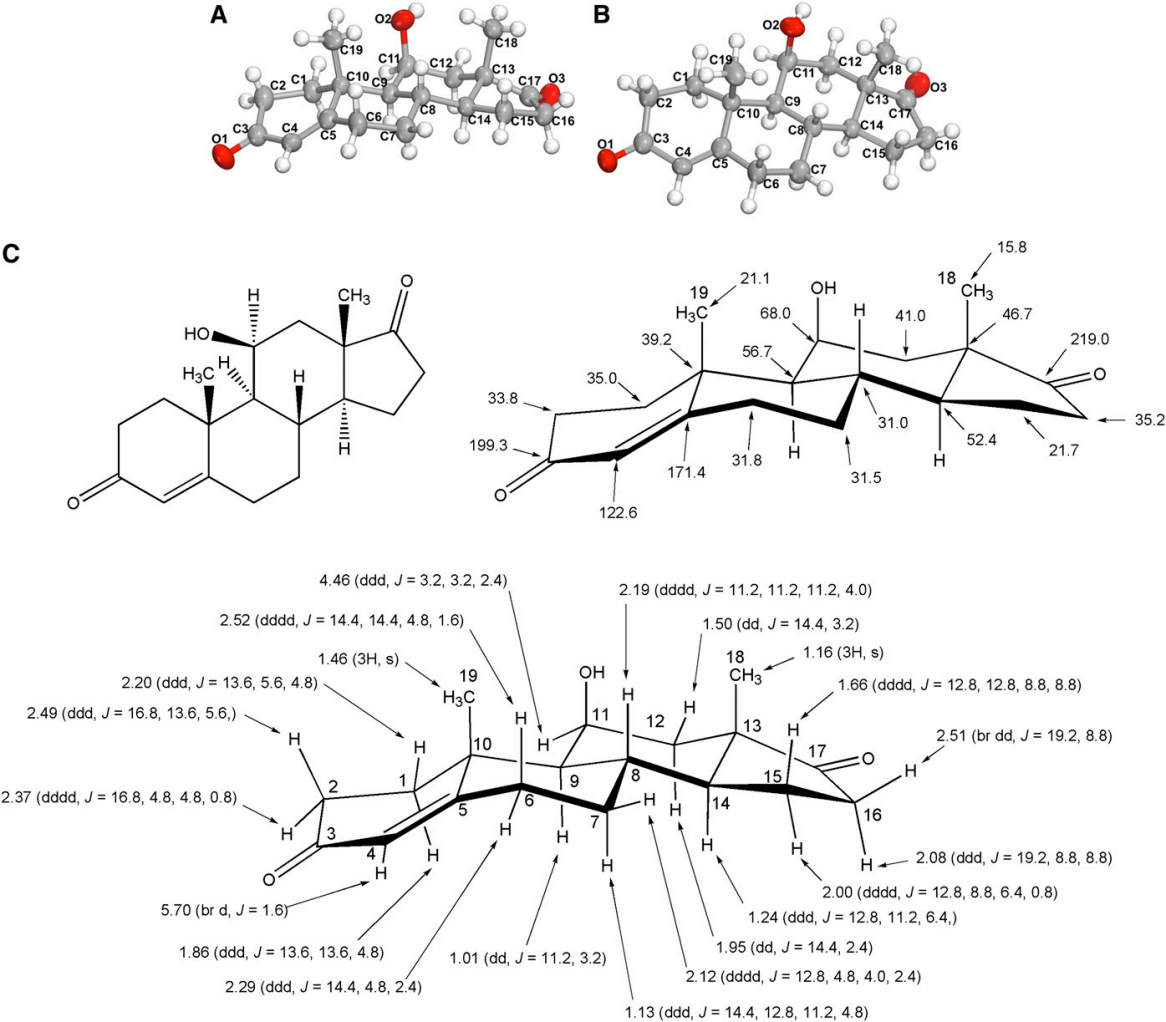
The molecular structure of the steroid-17,20-desmolase reaction product identified as 11β-hydroxyandrosten-3,17-dione. An Oak Ridge Thermal Ellipsoid Plot (ORTEP) view of the molecule with atomic labeling (thermal ellipsoids are drawn at 50% probability) (See also supplementary Table II for crystallographic details and supplementary Table IV for atomic coordinates, bond angles, and bond lengths)Oxygen atoms are in red, carbon atoms in grey, and hydrogen atoms in white. A: Side view of the molecule. B: Top view of the molecule. C: Standard chemical formula of the molecule (top left), stereochemical formula of the molecule displaying ^13^C NMR shifts (ppm) (top right), and stereochemical formula of the molecule displaying ^1^H NMR shifts (ppm) (bottom). See supplementary text and supplementary Table III for further description.

The steroid product formed by whole cells and cell extracts of *C. scindens* ATCC 35704 was extracted and purified by a combination of TLC and HPLC. The androgen exhibited an R_f_ of 0.78 on TLC plates using solvent A. The androgen had a retention time of 43.9 min on a semi-preparative C_18_ reversed-phase HPLC column as compared with cortisol, which eluted at 35.2 min. Reaction product was recrystallized from ether-*n*-hexane to give colorless needles (mp, 197–199°C; lit*197–199°C) (21). The structural determination was performed by a combination of techniques using HR-MS, ^1^H and ^13^C NMR, and X-ray diffraction analysis. HR-MS of the purified compound showed the deprotonated molecule [M-H]^−^ at *m/z* 301.18038, which is entirely identical with the calculated mass of 301.18037 (**Fig. 1**). The molecular structure of the steroid-17,20-desmolase product as 11β-OHA was determined unambiguously by ^1^H and ^13^C NMR and X-ray diffraction analysis of crystals of the steroid product (**Fig. 2**; supplementary Tables II–IV). We did not detect any accumulation of 11β-hydroxytestosterone or 11β-hydroxyepitestosterone from either whole cells or cell extracts of this bacterium. In cell extracts, the reaction proceeded under strictly anaerobic conditions and was not stimulated by NAD^+^/NADP^+^ suggesting a novel steroid-17,20-desmolase/oxidase catalyzing this biotransformation. For more structural details, see supplementary text.

### Identification of cortisol-regulated genes in *C. scindens* ATCC 35704 using RNA-Seq technology

**Fig. 3.**
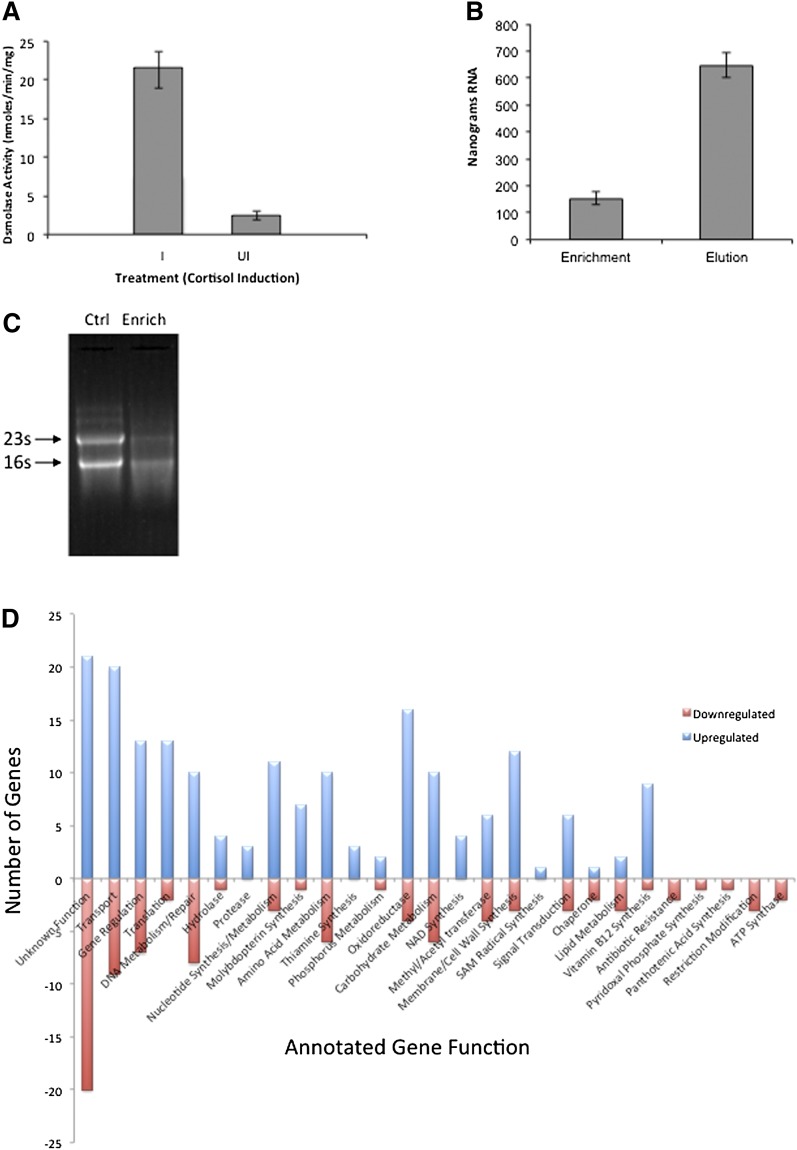
Measurement of cortisol induction, mRNA purification, and effect of cortisol induction on transcriptome of *C. scindens* ATCC 35704. A: Prior to isolation of mRNA for RNA-Seq, whole cells were measured for steroid-17,20-demolase activity by measurement of 11β-OHAD formation (n = 3). B: One microgram of total RNA was subjected to bead-capture hybridization (see Materials and Methods for details). We observed ∼80% removal of total RNA (Elution) by streptavidin bound magnetic beads coated with biotinylated oligonucleotides designed to bind 16s rRNA and 23s rRNA molecules. C: RNA gel demonstrating reduction in intensity of rRNA bands following bead-capture hybridization. Control (Ctrl) sample (10 μg total RNA) was subjected to bead-capture hybridization with beads lacking biotinylated capture oligos. Enrichment (Enrich) fraction containing enriched mRNA was sequenced. D: Results of RNA-Seq experiments comparing transcriptome data from control cells grown in BHI without cortisol and cortisol-induced cells. Upregulated genes are represented in blue, downregulated in red. Gene annotations from BLAST were used to group genes by general function (see supplementary Dataset I).

In previous studies we reported that steroid-17,20-desmolase activity was characteristic of *C. scindens* ATCC 35704, the type strain (6). However, we detected this activity in only three out of 31 strains of 7α-dehydroxylating human gut bacteria, many of which were identified as *C. scindens* by 16s rDNA gene sequencing (supplementary Table V). We chose *C. scindens* ATCC 35704 for identification of cortisol-inducible genes due to the presence of steroid-17,20-desmolase and 20α-HSDH activity and a genome sequence on which to scaffold RNA-Seq data. In order to use RNA-Seq as a method for identifying cortisol-inducible genes in *C. scindens* ATCC 35704, it was necessary to develop a method to enrich the mRNA, as it represents only about 1–5% of the total cellular RNA of bacteria. In this regard, we designed oligonucleotides along the length of the 16s rRNA and 23s rRNA molecules based on genomic sequences from *C. scindens* ATCC 35704 and *C. scindens* VPI 12708 (supplementary Table I) (22). Oligonucleotides were synthesized with biotinylated 3′ TEG spacers allowing for magnetic capture-hybridization of rRNA on Dynabeads linked to streptavidin (23). Before mRNA purification, we measured steroid-17,20-desmolase activity to verify cortisol induction (**Fig. 3A**). Quantification of the mRNA fraction (enrichment fraction) as well as the RNA eluted from magnetic beads coupled to our custom biotinylated rRNA capture oligos following capture (elution fraction) showed that our method removed ∼80% of the total RNA. Diminution of rRNA band intensity was observed between control total RNA (Ctrl), which used the same procedure as the mRNA enrichment sample (Enrich) except that capture oligos were omitted (Fig. 3B, C). Sequencing of the cDNA libraries resulted in around 300,000 reads using this sequencing technology for each library (cortisol-induced and uninduced control). Filtering of the ribosomal and transfer RNA sequences from these data showed that our rRNA depletion method removed about 90% of the rRNA from the libraries. However, considering that such sequences usually comprise ∼95–99% of the total extracted RNA, sequenced libraries still ended up with a 9:1 ratio of rRNA to non-rRNA sequence reads, with a final number of non-rRNA reads of 28,858 for the uninduced library and 27,701 for the cortisol-induced library. A combination of MEGAClear spin column and a pyrosequencing process allowed the removal of small RNAs (<100 bp). Only about 300 reads matched known tRNA sequences.

We have previously reported the characterization of the *baiBCDAFGHI* operon in *C. scindens*, which encodes genes involved in bile acid-inducible 7α/β dehydroxylation (1). We expected that if our method for isolating mRNA were valid, we should expect to see these genes significantly upregulated in cholic acid-treated cells. Experiments with 50 μM cholic acid induction were performed as described for cortisol (see Materials and Methods). Indeed, the *bai* operon was upregulated 452-fold as compared with control cells (supplementary Fig. I).

### Identification of a cortisol-inducible operon in *C. scindens* ATCC 35704

**Fig. 4.**
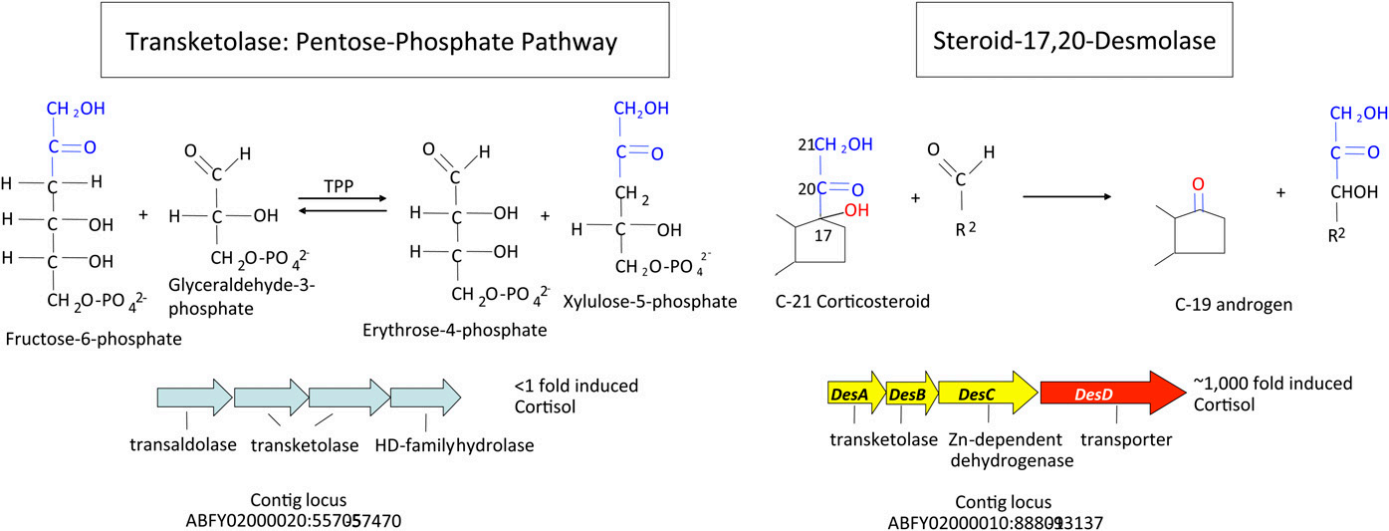
RNA-Seq data suggests a hypothesis for the mechanism of steroid-17,20-desmolase. We detected a cluster of four genes upregulated 1,000-fold by cortisol. This operon contains two genes encoding the N- and C-terminal subunits of a putative transketolase. The *C. scindens* ATCC 35704 genome contains additional transketolase genes which are not upregulated by cortisol and which display higher sequence identity with other transketolases found in nr BLASTP database (99–91% vs. 45–41% in first five hits; E-values 0.0 vs. 7e^−71^ to 4e^−60^). A comparison of the structure of the side chain removed by steroid-17,20-demolase, and transketolation reactions in the pentose-phosphate pathway, suggests that steroid-17,20-desmolase may proceed by TPP-dependent transketolation. HD, haloacid dehalogenase.

The genome (4.336 kbp) of *C. scindens* is estimated to encode 3,995 genes, of which a total of 186 genes were upregulated (2- to 1,000-fold) while 97 genes were downregulated by the addition of cortisol to growing cultures of *C. scindens* ATCC 35704. We grouped the data according to number of genes by function (Fig. 3D; supplementary Dataset S1). The most interesting genomic region upregulated in response to cortisol is an operon consisting of four open reading frames (upregulated ∼1,000-fold) two genes of which were annotated as N-terminal subunit (*desA*) (EDS07885.1) and C-terminal subunits of transketolase (*desB*) (EDS07886), the third gene encoding a putative zinc-dependent dehydrogenase (*desC*) (EDS07887), and the fourth a hypothetical sodium-dependent symporter (*desD*) (EDS07888) (**Fig. 4**). We also identified a gene apart from this operon encoding a putative ABC-type multidrug transport protein (EDS07925.1) that was upregulated 500-fold by cortisol that may represent an androgen exporter (supplementary Dataset S1). BLAST searches of *desAB* showed only 34% amino acid identity with other transketolases in the database. In contrast, three other genes encoding transketolase in the genome shared 85–97% amino acid identity with other annotated transketolases. Importantly, a BLAST search of the genome of *C. scindens* VPI 12708 (unpublished data), which lacks detectable cortisol-inducible steroid-17,20-desmolase activity, indicates that this gene cluster is not present in this organism. Similar BLAST searches of *Clostridium hylemonae* DSM 15053 (NZ_ABYI02000000) and *Clostridium hiranonis* DSM 13275 (NZ_ABWP01000000), both of which have bile acid 7α-dehydroxylating activity (21, 22), but lack detectable steroid-17,20-desmolase activity, also lack the *desABCD* genes (supplementary Table V).

At least 10 genes encoding enzymes involved in the biosynthesis of thiamine pyrophosphate (TPP) were induced from 3- to 500-fold (supplementary Dataset S1). All known transketolases require TPP as a cofactor. The two-carbon side chain of cortisol and the two-carbon group removed from a ketose donor during transketolation are identical suggesting the possibility that steroid-17,20-desmolase proceeds by a TPP-dependent mechanism (Fig. 4). Several genes encoding enzymes in the pentose-phosphate pathway were also upregulated by cortisol induction including: xylulose-5-phosphate isomerase (400-fold), ribulose-5-phosphate isomerase (276-fold), and ribose-5-phosphate isomerase (10-fold) (supplementary Dataset S1). This is expected if the two-carbon side chain of cortisol is feeding into the pentose-phosphate pathway. Fifteen genes for vitamin B_12_ biosynthesis were upregulated by cortisol (supplementary Dataset S1). The physiological function of B_12_ in *C. scindens* cortisol metabolism is currently unknown.

### The *desC* gene encodes an adrenocorticosteroid 20α-HSDH

**Fig. 5.**
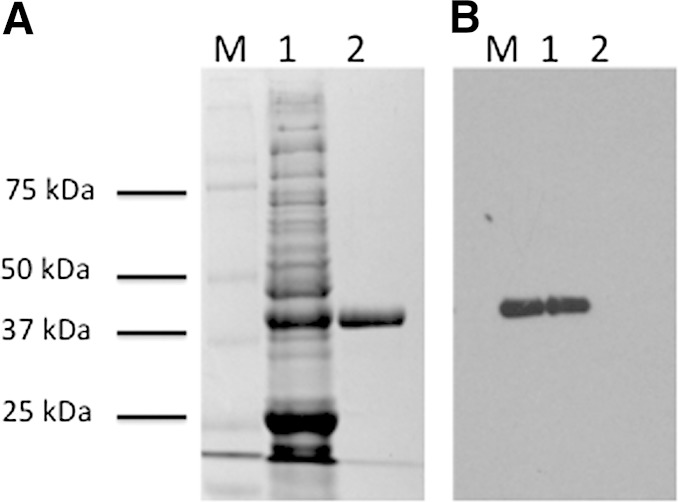
SDS-PAGE and Western immunoblot of recombinant DesC. A: SDS-PAGE. B: Western blot. M, protein marker; 1, crude extract of *E. coli* BL21(DE3)RIL after expression of recombinant DesC-ST; 2, purified recombinant DesC-ST after Strep-Tactin affinity chromatography.

It has been previously reported that *C. scindens* ATCC 35704 encodes steroid-inducible 40 kDa 20α-HSDH (7). Indeed, this enzyme has been purified and characterized, though the gene encoding this enzyme has yet to be identified (7, 8). An N-terminal sequence was previously reported for the 20α-HSDH; however, characterization of this and a second gene with matching N-terminal sequences showed that these genes encode enzymes with GADPH activity (supplementary text). Bioinformatic analysis of the *desC* (EDS07887.1) gene indicated that it encodes an NAD(P)-dependent oxidoreductase, M_r_ 38.5 kDa, induced 1,000-fold by cortisol, making this gene product a good candidate for the 20α-HSDH. The desC gene was cloned into pSport1 as an N-terminal streptavidin-tagged (ST) recombinant protein and purified by Strep-Tactin affinity chromatography to apparent electrophoretic homogeneity with an M_r_ of 40 kDa (**Fig. 5**).

We used several different glucocorticoid substrates to determine the specificity of this enzyme. We observed NADH-dependent reductive activity with cortisol (11β,17α,21-trihydroxypregn-4-ene-3,20-dione), but not 20α-cortisol (11β,17α,20α,21-tetrahydroxypregn-4-en-3-one), and 20β-cortisol (11β,17α,20β,21-tetrahydroxypregn-4-en-3-one) was not a substrate for this enzyme. When we tested each of these steroid substrates in the oxidative direction (NAD^+^) only 20α-cortisol was a substrate. This data suggests that the *desC* gene encodes an NAD^+^-dependent 20α-HSDH. The recombinant desC-ST showed an apparent *K_m_* of 5.35 μM and *V_max_* of 126 nmol/min/mg protein for cortisone, and an apparent *K_m_* of 1.46 μM and *V_max_* of 30 nmol/min/mg for cortisol in the presence of NADH. The apparent *K_m_* and *V_max_* for 20α-hydroxy cortisone was determined to be 4.54 μM and 24 nmol/min/mg protein, respectively. Substrates lacking a 17α-hydroxy group such as 11β,21-dihydroxypregn-4-ene-3,20-dione or those lacking a 21-hydroxy group such as 11β,17α-dihydroxypregn-4-ene-3,20-dione were not substrates. Finally, we did not detect 17α-HSDH activity against the end product of the steroid-17,20-desmolase reaction, 11β-OHA. These data are consistent with characteristics determined for the purified native 20α-HSDH isolated from this bacterium which recognize adrenocorticosteroids with 17α,21-dihydroxy groups (7, 8).

### Phylogenetic analysis of *desC*

**Fig. 6.**
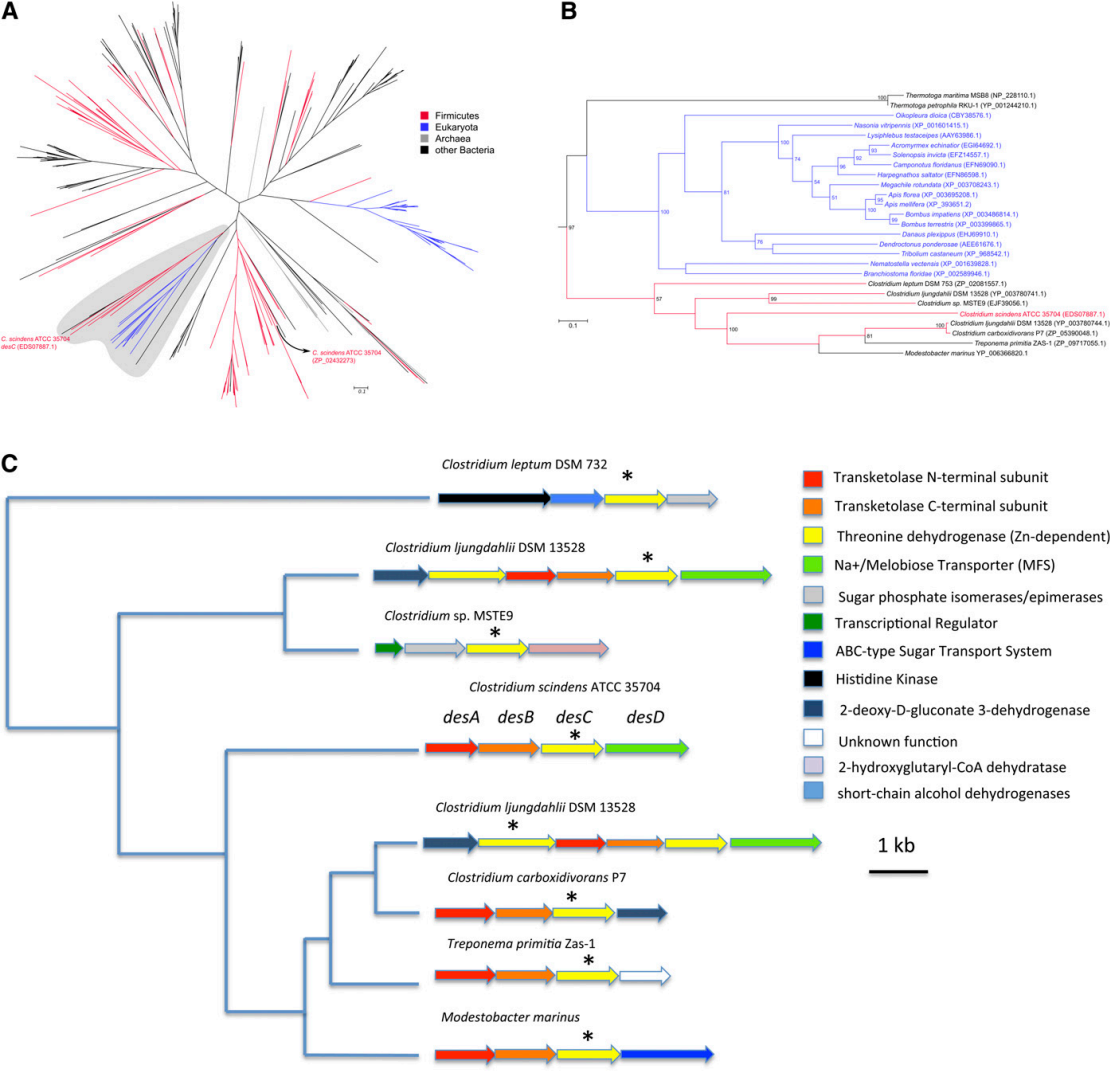
Maximum likelihood phylogenetic tree of *desC*. Values on nodes represent bootstrap support (only 50 or higher shown). A: Overall tree of 341 sequences from the second step of the analysis (see main text for details). B: Details of the region of the tree containing *C. scindens*’ *desC* [shaded area in panel (A)]. Colors reflect selected taxonomic groups, as depicted in the in-figure key. C: Organization of genes surrounding *desC* in shaded region of (B). Colors reflect gene function, as described in the in-figure key.

Annotation of the *desC* gene product suggests this gene evolved from threonine dehydrogenase (EC 1.1.1.103). We performed an extensive phylogenetic analysis to try to better understand the distribution of this gene in nature, and in particular the gut. Analysis of strains of *C. scindens* and other species of bile acid 7α-dehydroxylating bacteria suggest the *desC* gene is rare even in strains of *C. scindens* (supplementary Table V). The first step in the phylogenetic analysis of DesC, involving 2,045 sequences, identified a subtree of 341 sequences that contained *C. scindens*’ DesC and its nearest neighbors (**Fig. 6A**). Thorough analysis of these 341 sequences revealed the phylogenetic placement of DesC among sequences from a few other clostridia, one Actinobacteria (*Modestobacter*), one Spirochaetes (*Treponema*), a group of Metazoa (mostly insects), and two *Thermotoga* species, with the high bootstrap support value of 97 (Fig. 6B). All other Firmicutes sequences present in this subtree are distant from *C. scindens*’ DesC, including another *C. scindens* sequence (ZP_02432273, also from strain ATCC 35704). This sequence presents a much stronger match (E-value of 0) to the threonine dehydrogenase-like domain cd08234 than DesC (E-value 7e-63), and it is also placed in a group containing many other Firmicutes bacteria. Finally, if we examine genes surrounding *desC*-like genes in the cluster of microbial genes close to *C. scindens*’ *desC* and found a high degree of conservation of the transketolase subunits (corresponding to *desAB* in *C. scindens*) and the Na^+^/melibiose transporter (*desD*), hypothesized to encode a cortisol transport protein (Fig. 6C). Each of the genes in this cluster is associated with organisms that live in anaerobic environments, including the gut of animal species (24–27).

## DISCUSSION

*C. scindens* and a small number of species belonging to the genus *Clostridium* are responsible for significant alterations in the human bile acid pool composition through bile acid 7α/β dehydroxylation (1). Bile acids serve as natural ligands, with varying affinities, for nuclear receptors (farnesoid X receptor, pregnane X receptor, Vitamin D) and the G protein-coupled receptors TGR5 and SIP_2_ (28–32). Secondary bile acids, products of gut microbial metabolism, are the most potent activators of TGR5 (33, 34). Through activation of these receptors, bile acids regulate their own synthesis, conjugation, transport, and detoxification (reviewed by 30, 31), and are important regulators of lipid, glucose, and energy homeostasis (35, 36). Furthermore, bile acids play an important role in maintaining intestinal barrier function as antimicrobial agents in the small bowel (37, 38) and inducers of antimicrobial peptides (39). Perturbations in the biliary bile acid pool composition can be indicative of hepatogastrointestinal diseases such as fat malabsorption (40), gallstones (3), gastrointestinal cancers (41), and possibly type II diabetes (42). Recent studies also illustrate the important role gut microbes play in the levels and profiles of bile acids in various tissues of the body including: liver, kidney, plasma, serum, and heart (43). The physiological significance of these alterations in bile acid profiles in host organs such as heart and kidney is unknown. However, decades of research strongly suggest that secondary bile acids are associated with several diseases of the gastrointestinal system including: cancers of the colon, esophagus, and biliary duct (reviewed by 41, 44), as well as cholesterol gallstone disease in select patients (4).

In the current study, we have unambiguously demonstrated the presence of a 17-oxo group, identifying the end product of steroid-17,20-desmolase as 11β-OHA (Fig. 2). 11β-OHA is also known to be a primary adrenal steroid in the host, produced at a rate of 1.5 mg/day, slightly less than the rate of androstenedione formation (2.3–3.3 mg/day) (45). The function(s) of 11β-OHA in the human host is largely unknown (46, 47). However, it is known that 11β-OHA derived from metabolism of cortisol by gut microbes is reabsorbed into the bloodstream and excreted in urine (48).

**Fig. 7.**
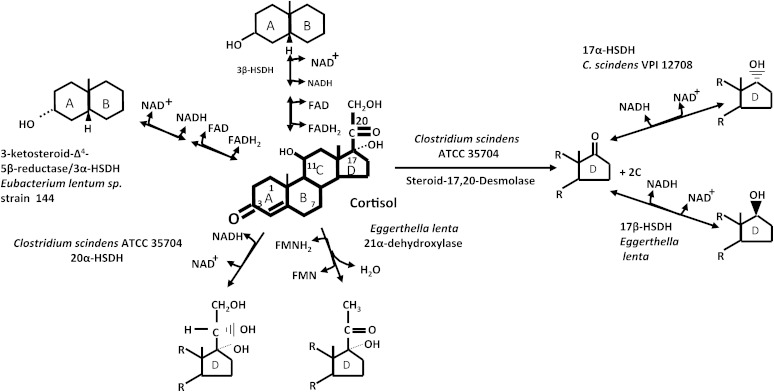
Cortisol metabolism by the human gut microbiome. Members of the human gut microbiome are capable of reducing, and epimerizing the 3-oxo group, reducing the Δ^4^-bond, oxidation/reduction of the 20-oxo group, removing the side-chain by steroid-17,20-desmolase, 21α-dehydroxylation, and epimerizing the 17-oxo group of 11β-OHAD.

There have been very few studies that have quantified glucocorticoids and their metabolites in human stool. Tracer studies of [^14^C]cortisol in humans suggest that only small quantities enter human bile, and that 90% of the radioactivity was found in urine and feces by 72 h (49, 50). Animals vary in the amounts of glucocorticoids secreted into the gut, and levels in stool are a marker for stress in animals under captivity (51). It is not presently known what the effect of 11β-OHAD is on the host, or for that matter other microbes. It has been reported that some intestinal microbes possess signal-transduction systems which respond to host hormones; a kind of inter-kingdom signaling (52). To date, these observations have been made in well-studied human pathogens. However, whether the biotransformation products of host steroids activate signaling pathways in members of the normal gut microbiota is unknown. We do know that other members of the resident microbiota form multiple metabolites from 11β-OHAD produced by *C. scindens*. The gut microbiota are capable of reducing the 17-oxo group of 11β-OHA to either the α- or β-configuration generating 11β-hydroxy-epitestosterone or 11β-hydroxy-testosterone, respectively (53, 54). In addition, some members of the gut microbiota can reduce the Δ^4^ bond in ring A to either 5α- or 5β-configuration resulting in *trans* or *cis* A/B rings, and can reduce the 3-oxo group to either 3α- or 3β-hydroxy groups (**Fig. 7**) (2). Thus steroid biotransformation products from one microbe yield further metabolites through the metabolic activity of other members of the human microbiome (Fig. 7). Microbial biotransformation products of host bile acids and steroids may play physiological roles in the host-microbiome axis.

**Fig. 8.**
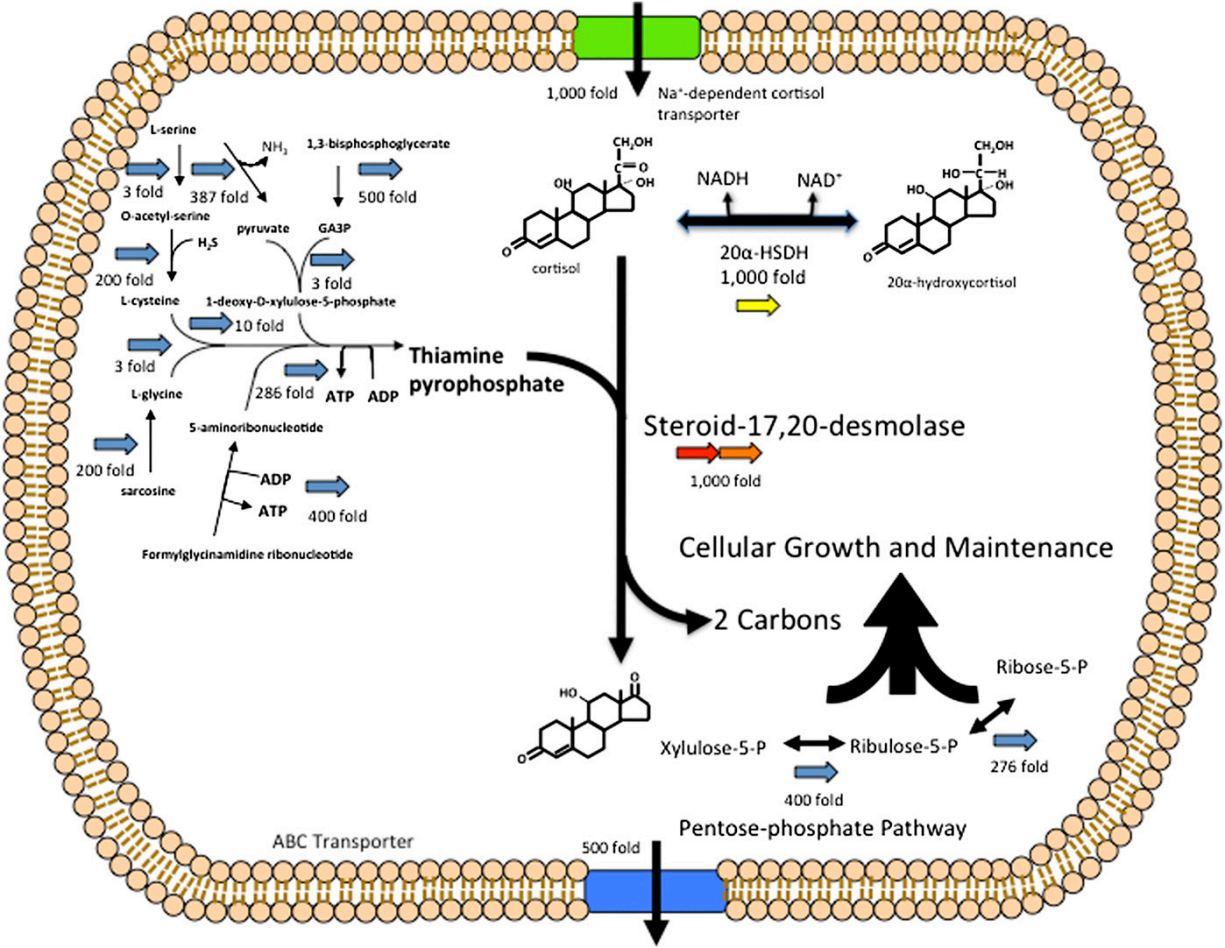
Model for cellular function of steroid-17,20-desmolase. Functional characterization of the *desC* gene (yellow) product supports our hypothesis that the transketolase encoded by the *desAB* genes (red, orange) encode steroid-17,20-desmolase, which proceeds by transketolation. Transketolases are TPP-dependent, and we observe an upregulation of genes involved in TPP synthesis. The *desD* gene (green) is predicted to encode a cortisol transport protein, and an ABC-type transporter was identified (500-fold induction; blue) which could serve to pump 11β-hydroxyandrostenedione out of the cell. We also observed several pentose phosphate pathway genes upregulated, suggesting that the two-carbon side chain might enter the pentose phosphate pathway. We hypothesize that the 20α-HSDH encoded by the *desC* gene regulates flux of the two-carbon fragment into the pentose phosphate pathway, as 20α-hydroxycortisol is not a substrate for steroid-17,20-desmolase.

In mammalian tissues, side-chain cleavage of steroids requires molecular oxygen-dependent monooxygenases (9). Side-chain cleavage of cortisol under anaerobic conditions by the gut microbiome has been known since 1959 (48, 55, 56); however, a mechanism has not been suggested until now. We hypothesize that the *desAB* genes encode the steroid-17,20-desmolase/oxidase and that this reaction proceeds by transketolation (Fig. 4 and **Fig. 8**). Previous work reported that TPP addition stimulates side-chain cleavage in cell extracts of *C. scindens* ATCC 35704 (7). Indeed, we observed a marked induction by cortisol of a number of genes involved in the biosynthesis of TPP (Fig. 8).

We have identified and characterized a cluster of coexpressed genes induced by cortisol, one of which encodes a 20α-HSDH. Reduction of the 20-oxo group to either α- or β-configuration blocks side-chain cleavage of C_21_-steroids by *C. scindens* (23). If the two-carbon fragment of cortisol is shuttled into the pentose-phosphate pathway, as our data suggests, it is possible that 20α-HSDH acts as a metabolic rheostat, regulating carbon flow (Fig. 8). A similar mechanism may be involved in the bile acid 7α-dehydroxylation pathway found in some intestinal clostridia, including *C. scindens* (1). Oxidation of the 7α-hydroxy group of cholic and chenodeoxycholic acid by 7α-HSDH inhibits bile acid 7α-dehydroxylation. These steroid HSDHs may act as “switches”, regulating entrance into steroid biotransformation pathways based on the NAD^+^/NADH ratio in the cell. The same phenomenon occurs in mammalian tissues expressing, for instance, AKR1C1, a human 20α-HSDH, whose function is to regulate the levels of progesterone over short time scales (10).

The human superorganism contains 20α-HSDH genes of both mammalian and microbial origin, which do not share common ancestry. Our phylogenetic analyses of *desC* show this gene as a very rare occurrence in clostridia and present only in five *Clostridium* species other than *C. scindens* ATCC 35704 (all of them distantly related to *C. scindens*). This suggests that *desC* is a novel ortholog derived from duplication (of an ancestral gene encoding a threonine dehydrogenase-like protein) followed by functional divergence and either gene loss in most clostridia or transfer from one species to a few select other species. There is also conservation of genes in the *desABCD* operon from *C. scindens* ATCC 35704 in organisms whose “*desC*” genes are most closely related, suggesting this operon may have passed in toto*.* Alternatively, this gene, and operon, could have been transferred to these organisms from a currently unknown source. Interestingly, multiple strains, which by 16s rDNA gene analysis were identified as *C. scindens*, were found to lack steroid-17,20-desmolase activity. This includes *C. scindens* VPI 12708 (formerly *Eubacterium* sp. strain VPI 12708) from which most of our knowledge of the bile acid 7α-dehydroxylation pathway has been elucidated (1). Interestingly, *C. scindens* VPI 12708, but not *C. scindens* ATCC 35704, expresses a 17α-HSDH, suggesting some niche partitioning of host glucocorticoid metabolism within a species of bile acid 7α-dehydroxylating bacteria (54). Reduction of oxo-groups by bacteria may in part serve to regenerate NAD^+^ necessary for fermentation. Host steroids and bile acids can therefore serve as electron sinks.

We now possess tools in the scientific community to uncover the relationship between our resident microbiota and the host. The “omics” revolution allows us to identify and quantify thousands of metabolites (metabolomics), and the genes encoding enzymes (genomics), the level at which genes are transcribed (transcriptomics), or the enzymes themselves (proteomics). However, this data is only as good as our ability to assign function or potential function to the genes. Steroids are important host signaling molecules. It has become clear that microbial metabolites of host steroids, such as deoxycholic acid, can have significant physiological and pathophysiological effects. Therefore, identification of the genes encoding enzymes responsible for gut microbial biotransformation of host steroids is important for future omics studies of host-gut microbe interactions in health and disease. The present data suggests that RNA-Seq analysis of inducible genes can aid in the discovery of genes in cultivated organisms known to carry out steroid-biotransforming reactions. It may also be possible to use this approach for gene discovery in mixed populations of microbes by comparing transcriptomes in the presence and absence of a particular gene inducer. Genes of interest can be cloned and characterized even from “unculturables” through a combination of transcriptomics and metagenomics.

## Supplementary Material

Supplemental Data
