# Supplementary material for: Clostridium scindens: a human gut microbe with a high potential to convert glucocorticoids into androgens
Source: J Lipid Res. 2013 Sep;54(9):2437–49. doi: 10.1194/jlr.M038869 (PMC3735941; doi:10.1194/jlr.M038869)
Supplement: Supplemental Data [file supp_54_9_2437__index.html]

Clostridium scindens: a human gut microbe with a high potential to convert glucocorticoids into androgens — Supplemental Data 

# *Clostridium scindens*: a human gut microbe with a high potential to convert glucocorticoids into androgens

## 

**Files in this Data Supplement:**

- SI Text - PDF file containing SI text, SI tables, SI figures.
- RNA Seq Dataset - RNA Seq Dataset of differential gene expression.
