# Supplementary material for: Clostridium scindens: a human gut microbe with a high potential to convert glucocorticoids into androgens
Source: J Lipid Res. 2013 Sep;54(9):2437–49. doi: 10.1194/jlr.M038869 (PMC3735941; doi:10.1194/jlr.M038869)
Supplement: Supplemental Data [file supp_M038869_jlr.M038869-1.pdf]

SUPPORTING INFORMATION

SI Results and Discussion

Identification of 20α-HSDH gene. A 20α-HSDH was previously purified from *C. scindens* ATCC 35704 and an N-terminal amino acid sequence determined which shares 100% identity with ZP_02431184 and 71% with the N-terminus of EDS07660.1 from the *C. scindens* ATCC 35704 genome (1). BLAST analysis of these ORFs suggested they encode glyceraldehyde-3-phosphate dehydrogenases (GAPDH). Genome-wide transcriptomic data (RNASeq) between cortisol-induced and uninduced control cells show that EDS07660.1, but not ZP_02431184, is induced 500 fold by cortisol (Dataset S1; Fig. S2). To determine whether either of these genes encoded an enzyme with 20α-HSDH or GAPDH activity, we cloned them into a pSport1 vector, overexpressed and purified them. We detected strong GADPH activity by both of these gene products, and no activity toward cortisol, cortisone, or 20α-cortisone (see SI Methods below). Examination of the final protein fraction isolated by Krafft (1989), when separated by 2D-Gel showed two proteins of similar quantity, and size, but different pI (1, 2). We conclude that GADPH (ZP_02431184) was a major contaminant, and perhaps, the N-terminus of the 20α-HSDH is methylated and refractory to sequencing. Data presented in the main text demonstrates that the *des*C gene (EDS07887.1) encodes an enzyme with 20α-HSDH activity with characteristics consistent with those reported by Krafft (1989).

Structural characterization of steroid-17,20-desmolase product.

In the ^1H NMR spectrum of the androgen, a pair of singlets appearing at 1.16 and 1.46 ppm were assigned to the three protons at C-18 and C-19 methyl groups, providing evidence for the structure of the C_{19} androstane moiety (Figure 2C, Table S3). A signal appearing at 5.70 ppm as a broad doublet was assigned to the 4-H. These observations indicated that the purified compound has the 3-oxo-Δ^4 structure in the A/B ring juncture. Furthermore, the occurrence of the two quaternary ^{13}C signals at 199.3 and 171.4 ppm and the tertiary ^{13}C signal at 122.6 ppm in the ^{13}C NMR spectrum of the compound indicated the presence of the conjugated enone moiety. The 2D HMBC spectrum of the compound showed correlation peaks between 19-H_3 vs C-5 and 4-H vs C-5 strongly indicating the presence of the Δ^4-bond. Essentially identical ^1H and ^{13}C-NMR characteristics were observed in the spectra of 11β-OHA. The stereochemistry of the steroid-17,20-desmolase reaction product was unambiguously determined by X-ray analysis. An ORTEP view of the molecule with atomic labeling (thermal ellipsoids are drawn at 50% probability) is shown in Figure 2A,B of the main text. There are two crystallographically independent molecules in the unit cell, which show no significant geometrical or stereochemical differences. The bond distances and bond angles have the expected values for these kinds of steroids. Selected bond lengths and bond angles are presented in Table S4. In particular, the enone O=C-C=C fragment in ring A of the two independent molecules are almost anti-periplanar with a small dihedral angle of 1.56° and 3.22°, respectively. The bond distances are 1.238(6) and 1.239(7) for O1-C3 (O4-C22), 1.463(7) and

1.455(7) for C3-C4 (C22-C23), 1.333(6) and 1.342(6) Å for C4-C5 (C23-C24) respectively. And the bond distances of O3-C17 (O6-C36) of the ketone group in ring D are 1.209(6) and 1.225(6) Å, respectively.

Identification of bile acid 7α-dehydroxylating clostridia with steroid-17,20-desmolase activity

To date, all known bile acid 7α-dehydroxylating bacteria are members of the genus *Clostridium* (3). The ability to side-chain cleave host corticosteroids is a capacity which defines the type strain of *Clostridium scindens*, indeed "scindens" means "to cut" (4). We tested 15 strains of *Clostridium scindens* for steroid-17,20-desmolase activity. Many of these strains were previously isolated and characterized in our lab (5, 6) and others (7, 8), as well as isolates recently isolated and identified in the lab of Dr. Fusae Takamine. We also tested the type strains of *C. hylemonae, C. hiranonis* and *C. sordellii,* in addition to a strain of *C. bifermentans* and 12 additional isolated from centenarians which have yet to be classified. Our results demonstrate that steroid-17,20-desmolase activity is so far constrained to *C. scindens*, and even then, we were only able to detect this activity in 3 (35704, S076, S077) out of 15 strains of this species tested (Table S5). This observation is important because it appears that *C. scindens* is a misnomer for many strains that share the threshold 16s rDNA sequence identity.

SI Methods

Cloning, overexpression, and characterization of ORFs.

Genes identified in the genome which corresponded with N-terminal sequences previously identified with 20α-HSDH were cloned into pSport1 vector using the

following cloning primers: pSportCTSBPEDS07660.1F 5’-CTGCAGAGGAGGTAATTGT- ACAATGGCAATTCAAATTGG-3’ and pSportCTSBPEDS07660.1R 5’-GGATCCTTATTTTTCGAACTGCGGGTGG-CTCCATTGCTGATGAATCTGGTCCATGTAT-TC-3’; pSportCTSBPZP0243-1184F 5’-CTGCAGAAACTTTAGGAGGAATTAATCATGGCA-3’, and pSportCT-SBPZP02431184R 5’-ATAGGATCCTTATTTTCGAACTGCGGGTGGCTCCAAG-CGCTAGCCAGTTCAGAGAAG-3’. Details regarding cloning, overexpression, and purification were followed as per methods in main text. Recombinant proteins were engineered with streptavidin C-terminal tags. Expression was verified by Western immunoblot with Strep Tag II Antibody. Glyceraldehyde-3-phosphate dehydrogenase activity was measured by continuous spectrophotometric assay at 340 nm. As a positive control, we used glyceraldehyde-3-phosphate dehydrogenase from Baker’s Yeast (Sigma-Aldrich, St. Louis, MO). Reactions were performed in 20 mM sodium pyrophosphate containing 1 mM dithiothreitol, 150 µM NAD, 50 µM DL-glyceraldehyde-3-phosphate (prepared according to the manufacturer, Sigma-Aldrich, St. Louis, MO).

SI References

1. Krafft, A.E., and P.B. Hylemon. 1989. Purification and characterization of 20α-hydroxysteroid dehydrogenase from *Clostridium scindens*. *J. Bacteriol.* **171**:2925-2932.
2. Krafft, A.E. 1989. Characterization of steroid-17,20-desmolase and 20α-hydroxysteroid dehydrogenase from *Clostridium scindens*. Doctoral Dissertation, pg 98.
3. Ridlon, J.M., D.J. Kang, and P.B. Hylemon. 2006. Bile salt biotransformations by human intestinal bacteria. *J. Lipid Res.* **47(2)**:241-259.
4. Winter, J., G.N. Morris, S. O'Rourke-Locascio, V.D. Bokkenheuser, E.H. Mosbach, B.I. Cohen, and P.B. Hylemon. 1984. Mode of action of steroid desmolase and reductases synthesized by *Clostridium "scindens"* (formerly Clostridium strain 19). *J. Lipid Res*. **25(10)**:1124-1131.
5. Wells, J.E., F. Berr, L.A. Thomas, R.H. Dowling, and P.B. Hylemon. 2000. Isolation and characterization of cholic acid 7alpha-dehydroxylating fecal bacteria from cholesterol gallstone patients. *J. Hepatol.* **32(1)**:4-10.
6. Doerner, K.C., F. Takamine, C.P. LaVoie, D.H. Mallonee, P.B. Hylemon. 1997. Assessment of fecal bacteria with bile acid 7α-dehydroxylating activity for the presence of *bai*-like genes. *Appl. Environ. Microbiol.* **63**:1185–1188
7. Takamine, F., and T. Imamura. 1995. Isolation and characterization of bile acid-inducible 7α-dehydroxylating bacteria from human feces. *Microbiol. Immunol.* **39**:11–18
8. Hirano, S., R. Nakama, M. Tamaki, N. Mauda, and H. Oda. 1981. Isolation and characterization of thirteen intestinal microorganisms capable of 7α-dehydroxylation of bile acids. *Appl. Environ. Microbiol.* **41**:737–745.

SI Figure Legends

Figure S1. Gbrowse representation of raw RNA-Seq reads under different conditions along the *bai* operon. The *baiBCDEAFGHI* operon is induced by bile acids such as cholic acid. We wanted to determine whether our mRNA enrichment was successful to identify inducible genes so we sequenced mRNA enriched from uninduced control cells (IU reads), cortisol-induced cells (Cort Reads), and cholic acid-induced cells (CA reads). Only cholic acid induction resulted in detection of mRNA reads in the *bai* operon.

Figure S2. Gene organization surrounding ORFs located from N-terminal Sequencing of purified 20α-HSDH. We located two genes corresponding to peptide derived from N-terminal sequence previously obtained from 20α-HSDH from *C. scindens* ATCC 35704 (SI reference 1). Both genes were cloned, overexpressed, and found to encode glyceraldehyde-3-phosphate dehydrogenases that did not have measurable 20α-HSDH activity. Contig accession number, base pairs, protein accession numbers, and relative expression levels (based on RNA-Seq experiments) given. * Experimentally verified in our lab.

Table S1. Sequence of biotinylated oligonucleotides used for rRNA-capture

16s rRNA Capture Oligonucleotides	
16s35704cap1	GCGTTACTGACTCCCATGGTGTGACGG-BioTEG
16s35704cap2	CTTGCGAACGTACTCCCCAGGTGGACTA-BioTEG
16s35704cap3	GCTTCGGTCTTATGCGGTATTAGCAGCC-BioTEG
23s rRNA Capture Oligonucleotides	
23s35704cap1	CCAGGGTAGCTTTTATCCGTTGAGCGA-BioTEG
23s35704cap2	GACAGTGCCCAAATCATTACGCCTTTCG-BioTEG
23s35704cap3	AACCTGTTGTCCATCGGCTACGGC-BioTEG
23s35704cap4	GGACATGGATAGATCACCCGGTTTCG-BioTEG
23s35704cap5	GACACCTCCGGATCAAAGGGTATTTGCC-BioTEG

See SI Methods for primer design

Table S2. Crystallographic Information

	Steroid-17,20-desmolase product
Formula	$(C_{19}H_{26}O_3)_2$
M	604.82
Crystal System	Orthorhombic
Space Group	$P2_12_12_1$
a (Å)	6.042(1)
b (Å)	20.241(5)
c (Å)	26.619(6)
V (Å^3)	3255.6(13)
Z value	4
D_c/g·cm^{-3}	1.234
F(000)	1312
μ(Mo-K_α)/cm^{-1}	0.815
No. Reflns Measured.	23027
No. Unique Reflns.	5939
No. Observed. (I>2σ)	4003
R_1 (I>2σ)	0.065
wR_2 (I>2σ)	0.093
Goodness of Fit	1.025

Table S3 ^{1}H and ^{13}C NMR chemical shifts of androgen derivative. [a]

Carbon no.	Androgen derivative			
	^{13}C		^{1}H	
	Type	Chemical shift	Configuration	Chemical shift
1	CH_2	35.0	α<br>β	1.86 (ddd, *J*=13.6, 13.6, 4.8 Hz)<br>2.20 (dddd, *J*=13.6, 5.6, 4.8 Hz)
2	CH_2	33.8	α<br>β	2.37 (dddd, *J*=16.8, 4.8, 4.8, 0.8 Hz)<br>2.49 (ddd, *J*=16.8, 13.6, 5.6 Hz)
3	C	199.3		
4	CH	122.6		5.70 (br d, *J*=1.6 Hz)
5	C	171.4		
6	CH_2	31.8	α<br>β	2.29 (ddd, *J*=14.4, 4.8, 2.4 Hz)<br>2.52 (dddd, *J*=14.4, 14.4, 4.8, 1.6 Hz)
7	CH_2	31.5	α<br>β	1.13 (ddd, *J*=14.4, 12.8, 11.2, 4.8 Hz)<br>2.12 (dddd, *J*=12.8, 4.8, 4.0, 2.4 Hz)
8	CH	31.0	β	2.19 (dddd, *J*=11.2, 11.2, 11.2, 4.0 Hz)
9	CH	56.7	α	1.01 (dd, *J*=12.2, 3.2 Hz)
10	C	39.2		
11	CH	68.0	α	4.46 (ddd, *J*=3.2, 3.2, 2.4 Hz)
12	CH_2	41.0	α<br>β	1.95 (dd, *J*=14.4, 2.4 Hz)<br>1.50 (dd, *J*=14.4, 3.2 Hz)
13	C	46.7		
14	CH	52.4	α	1.24 (ddd, *J*=12.8, 11.2, 6.4 Hz)
15	CH_2	21.7	α<br>β	2.00 (dddd, *J*=12.8, 8,8, 6.4, 0.8 Hz)<br>1.66 (dddd, *J*=12.8, 12.8, 8.8, 8.8 Hz)
16	CH_2	35.2	α<br>β	2.08 (ddd, *J*=19.2, 8.8, 8.8 Hz)<br>2.51 (br dd, *J*=19.2, 8.8 Hz)
17	C	219.0		
18	CH_3	15.8		1.16 (s)
19	CH_3	21.1		1.46 (s)

[a] Measured in $CDCl_3$ at 800 MHz in ^{1}H NMR and 200 MHz in ^{13}C NMR; chemical shifts were expressed as δ ppm relative to Me_4Si; abbrev. used: s, singlet; d, doublet; values in parentheses to signal multiplicity and coupling constant (*J* in Hz).

Table S4. Atomic coordinates (a) and Selected bond lengths (Å) and bond angles (°) (b) for steroid-17,20-desmolase product Atomic coordinates for steroid-17,20-desmolase product

(a)

atom	x	y	z
O1	1.0347(7)	1.2754(2)	0.90103(13)
O2	0.4305(7)	1.0147(2)	1.00736(12)
O3	0.8640(7)	0.7992(2)	1.03505(12)
O4	0.1575(8)	1.0301(2)	0.60910(13)
O5	0.8031(6)	0.7680(2)	0.70171(13)
O6	0.3435(7)	0.5798(2)	0.77673(13)
C1	0.7702(9)	1.1502(2)	0.9765(2)
C2	0.7860(10)	1.2209(3)	0.9566(2)
C3	0.9311(8)	1.2245(2)	0.9112(2)
C4	0.9355(8)	1.1665(2)	0.8784(2)
C5	0.8214(8)	1.1115(2)	0.8879(2)
C6	0.8029(10)	1.0596(2)	0.8479(2)
C7	0.8542(10)	0.9905(3)	0.8679(2)
C8	0.7264(8)	0.9738(2)	0.9155(2)
C9	0.7588(8)	1.0282(2)	0.9556(2)
C10	0.6975(7)	1.0993(2)	0.9365(2)
C11	0.6708(8)	1.0109(2)	1.0074(2)
C12	0.7459(9)	0.9420(3)	1.0257(2)
C13	0.6990(8)	0.8888(2)	0.9867(2)
C14	0.8061(8)	0.9087(2)	0.9370(2)
C15	0.7981(10)	0.8452(2)	0.9052(2)
C16	0.8524(10)	0.7915(3)	0.9438(2)
C17	0.8110(8)	0.8221(2)	0.9948(2)

C18	0.4476(9)	0.8724(3)	0.9812(2)
C19	0.4468(9)	1.1077(3)	0.9250(3)
C20	0.4599(10)	0.8822(3)	0.6367(2)
C21	0.4432(12)	0.9494(3)	0.6111(2)
C22	0.2648(9)	0.9902(3)	0.6346(2)
C23	0.2321(8)	0.9829(2)	0.6885(2)
C24	0.3406(8)	0.9377(2)	0.7160(2)
C25	0.3228(10)	0.9383(2)	0.7725(2)
C26	0.2723(9)	0.8703(2)	0.7944(2)
C27	0.4317(8)	0.8161(2)	0.7748(2)
C28	0.4356(7)	0.8174(2)	0.7168(2)
C29	0.4947(7)	0.8861(2)	0.6936(2)
C30	0.5688(8)	0.7598(2)	0.6941(2)
C31	0.5007(9)	0.6924(2)	0.7154(2)
C32	0.4921(7)	0.6915(2)	0.7726(2)
C33	0.3454(8)	0.7485(2)	0.7911(2)
C34	0.3103(10)	0.7319(3)	0.8470(2)
C35	0.2874(10)	0.6555(3)	0.8466(2)
C36	0.3745(9)	0.6340(3)	0.7961(2)
C37	0.7264(9)	0.6911(3)	0.7963(2)
C38	0.7315(9)	0.9097(3)	0.7059(3)

(b)

O1-C3	1.238(6)	O1-C3-C2	121.1(4)
C3-C4	1.463(7)	O1-C3-C4	121.8(5)
C4-C5	1.333(6)	C2-C3-C4	117.0(4)
O2-C11	1.455(7)	C3-C4-C5	123.1(5)
O3-C17	1.209(6)	O3-C17-C13	125.7(4)
O4-C22	1.239(7)	O3-C17-C16	126.4(4)
C22-C23	1.455(7)	C13-C17-C16	107.9(4)
C23-C24	1.342(6)	O4-C22-C21	120.7(5)
O5-C30	1.439(6)	O4-C22-C23	122.4(5)
O6-C36	1.225(6)	C21-C22-C23	116.9(5)
		C22-C23-C24	122.7(5)
		O6-C36-C32	126.2(5)
		O6-C36-C35	125.5(5)
		C32-C36-C35	108.2(4)

Table S5: Identification of strains of bile acid 7α-dehydroxylating bacteria with steroid-17,20-desmolase activity

Bile acid 7α-dehydroxylating Strain	Steroid-17,20-desmolase Activity
Clostridium scindens ATCC 35704	+
Clostridium scindens S076	+
Clostridium scindens S077	+
Clostridium scindens 36S	−
Clostridium scindens Y1113	−
Clostridium scindens TH82	−
Clostridium scindens I10	−
Clostridium scindens KS11	−
Clostridium scindens KS31	−
Clostridium scindens MM12	−
Clostridium scindens MO321181	−
Clostridium scindens NT182	−
Clostridium scindens SA14	−
Clostridium scindens SY14	−
Clostridium hylemonae DSM 15053	−
Clostridium hiranonis DSM 13275	−
Clostridium bifermentans I-55	−
Clostridium sordellii Y67	−
Clostridium sordellii ATCC 9714	−
C132	−
C571	−
C592	−
E33	−
G11	−
HU6	−
C17	−
I102	−
I127	−
IB6	−
K163	−
O22	−

Figure S1

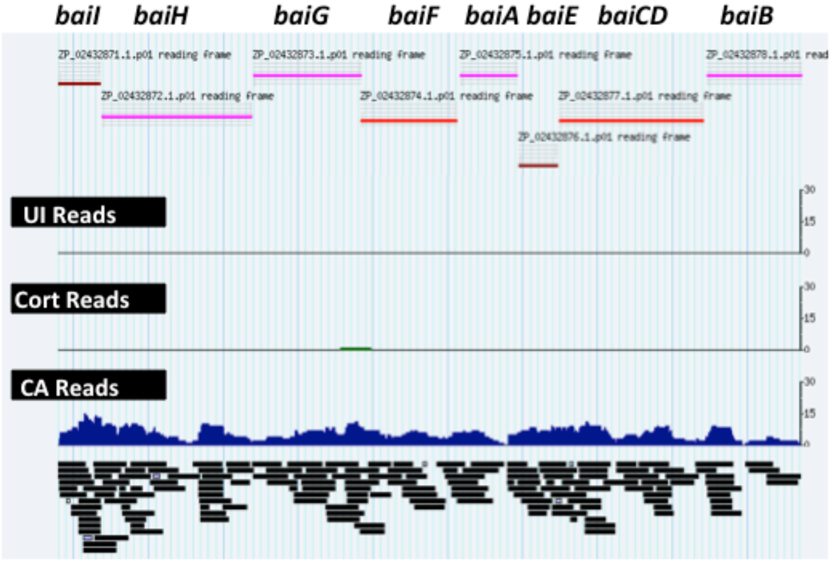

baiI
baiH
baiG
baiF
baiA
baiE
baiCD
baiB
UI Reads
Cort Reads
CA Reads
30
15
0

Figure S2

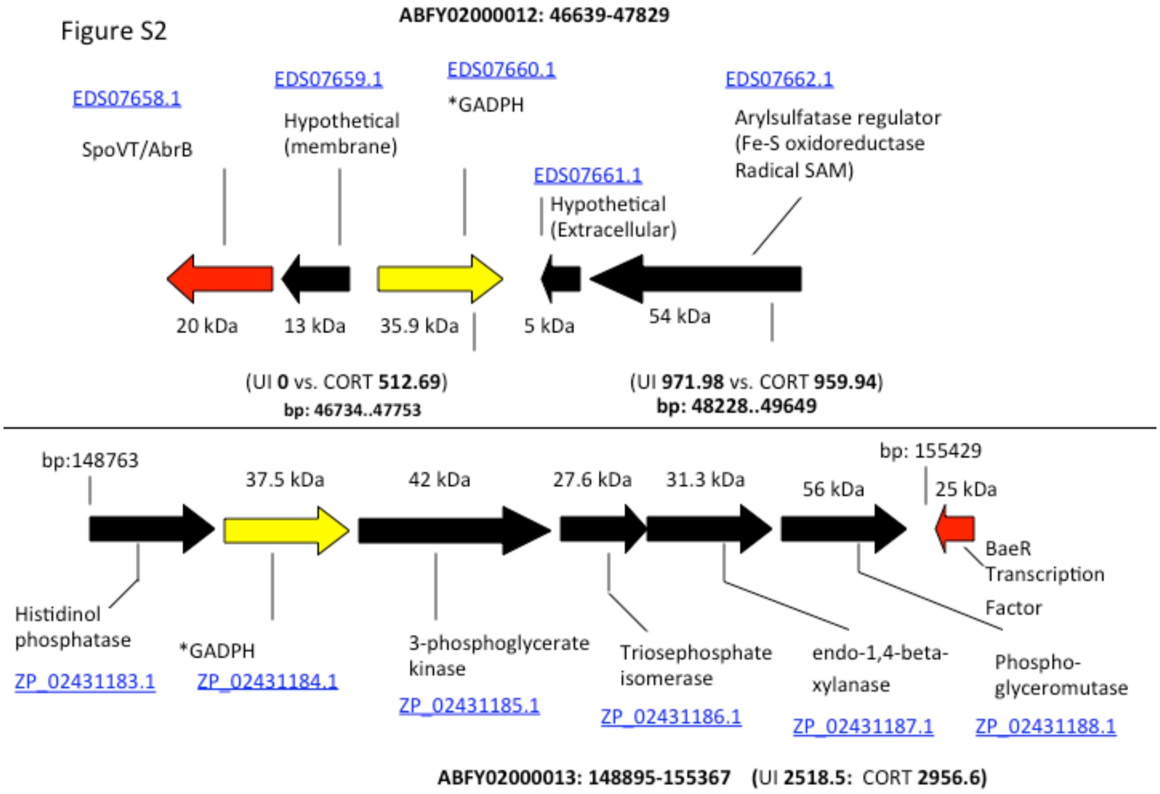
ABFY02000012: 46639-47829
EDS07658.1
SpoVT/AbrB
EDS07659.1
Hypothetical (membrane)
EDS07660.1
*GADPH
EDS07661.1
Hypothetical (Extracellular)
EDS07662.1
Arylsulfatase regulator (Fe-S oxidoreductase Radical SAM)
20 kDa
13 kDa
35.9 kDa
5 kDa
54 kDa
(UI 0 vs. CORT 512.69)
bp: 46734..47753
(UI 971.98 vs. CORT 959.94)
bp: 48228..49649
bp:148763
37.5 kDa
42 kDa
27.6 kDa
31.3 kDa
56 kDa
bp: 155429
25 kDa
BaeR Transcription Factor
Histidinol phosphatase
ZP_02431183.1
*GADPH
ZP_02431184.1
3-phosphoglycerate kinase
ZP_02431185.1
Triosephosphate isomerase
ZP_02431186.1
endo-1,4-beta-xylanase
ZP_02431187.1
Phospho-glyceromutase
ZP_02431188.1
ABFY02000013: 148895-155367 (UI 2518.5: CORT 2956.6)
